# Synthesis and Electrochemical Performance of Microporous Hollow Carbon from Milkweed Pappus as Cathode Material of Lithium–Sulfur Batteries

**DOI:** 10.3390/nano12203605

**Published:** 2022-10-14

**Authors:** Jun-Ki Kim, Yunju Choi, Euh Duck Jeong, Sei-Jin Lee, Hyun Gyu Kim, Jae Min Chung, Jeom-Soo Kim, Sun-Young Lee, Jong-Seong Bae

**Affiliations:** 1 Busan Center, Korea Basic Science Institute (KBSI), Busan 46742, Korea; 2 Jeonju Center, Korea Basic Science Institute (KBSI), Jeonju 54907, Korea; 3 Division of Plant Resources, Korea National Arboretum, Seoul 02455, Korea; 4 Department of Chemical Engineering, Dong-A University, Busan 49315, Korea; 5 Secondary Batteries Technology Center, Chungnam Techno Park, Cheonan 31035, Korea

**Keywords:** milkweed pappus, microporous hollow carbon, lithium–sulfur batteries

## Abstract

Microtube-like porous carbon (MPC) and tube-like porous carbon–sulfur (MPC-S) composites were synthesized by carbonizing milkweed pappus with sulfur, and they were used as cathodes for lithium–sulfur batteries. The morphology and uniformity of these materials were characterized using X-ray powder diffraction, Raman spectroscopy, scanning electron microscopy, transmission electron microscopy with an energy-dispersive X-ray analyzer, thermogravimetric analysis, and X-ray photoelectron spectrometry. The electrochemical performance of the MPC-S cathodes was measured using the charge/discharge cycling performance, C rate, and AC impedance. The composite cathodes with 93.8 wt.% sulfur exhibited a stable specific capacity of 743 mAh g^−1^ after 200 cycles at a 0.5 C.

## 1. Introduction

The rapid increase in energy requirements has led to the development of safe, clean, and renewable energy systems, including solar cells, fuel cells, and batteries. Lithium–sulfur (Li-S) batteries are being actively investigated as next-generation batteries that can overcome the disadvantage of the low energy density of commercial lithium-ion batteries. Sulfur is used as a cathode material in these batteries, and it has a high theoretical specific capacity of 1675 mAh g^−1^ and a high energy density of 2600 Wh kg^−1^ [1]. However, Li-S batteries experience problems including polysulfide dissolution, loss of sulfur, low utilization of the active area, nonconductivity, poor cycle life, capacity fading, and safety issues [2]. To achieve a high electrochemical performance of Li-S batteries, sulfur must be in intimate contact with conductors such as carbon or conducting polymers [3].

The batteries developed by Nazar et al. employed mesoporous carbon with a highly uniform pore structure for boosting battery efficiency. Sulfur was melted, and pores were uniformly filled with sulfur, thereby minimizing the surface area in direct contact with carbon [4]. Researchers have applied various carbon materials, such as amorphous carbon, carbon black [5,6], macro/meso/microporous carbon [7,8], carbon nanofibers [9,10], carbon nanotubes [11,12,13], graphite, graphene oxide, and reduced graphene oxide, to enhance the electronic conductivity of sulfur composites and inhibit the dissolution of polysulfides into cell electrolytes [14,15,16,17,18]. The carbonaceous materials that originate from biomaterials are being increasingly used to produce low-cost and high-performance en ergy storage devices as an efficient method of maintaining a clean and sustainable world [19,20,21,22,23]. In the past decade, the application of active carbon obtained from biomass, such as banana peels [24], bamboo leaves [25], soybeans [26], corn cobs [27], pomelo peels [28], shaddock peels [29], poplar catkins [30], and kapok fibers [31], fish industry waste [32] to Li-S batteries has been investigated. Our group examined the use of porous carbon derived from thiourea, calcium citrate [33], and garlic peels to Li-S batteries [34].

Milkweed (MW) is distributed throughout Korea, Japan, and China. The tubular seeds of MW have microporous hollow fibers, which are comprised of 55% cellulose and 18% lignin. Milkweed pappus has regular microfibers with a diameter of 17–20 μm. When this is heat treated at high temperature in an atmosphere, it maintains the shape of a tube of porous microfibers with a diameter of about 5 μm. These fibers can provide external and internal surfaces for electron transfer. MW pappus has a hollow fiber structure that provides a large interspatial area for oil sorption [35]. Lou et al. recently studied supercapacitor materials that used Metaplexis plants with MW-like structures as carbon electrode sources [36].

In the present study, to improve the electrical conductivity of sulfur in Li-S batteries, mesoporous, carbonized, conductive, and microtube-like porous carbon/sulfur (MPC-S) composites were synthesized by heating a mixture of carbonized MW fibers and sulfur. The electrochemical performance of the MPC-S cathodes for Li-S cells was investigated using cyclic voltammetry (CV), charge/discharge cycling performance, C rate, and AC impedance.

## 2. Experimental Methods

### 2.1. Preparation of Milkweed Pappus Carbon (MPC)

MW pods were collected from a local region (Kyeongkido, Korea). MW pappus with hollow microfibers was obtained by removing the seeds from the MW pods. MW pappus was thoroughly washed with ethanol and distilled water to remove dust and then dried at 90 °C for 24 h. The cleaned MW pappus was precarbonized at 500 °C for 1 h at a heating rate of 3 °C min^−1^ in a N_2_ atmosphere. Next, the MW pappus was placed in a tube furnace and heated to 800 °C at a rate of 3 °C min^−1^ in a N_2_ atmosphere for 1 h. The carbonized sample was washed in 0.1 M hydrogen chloride (HCl, ACS reagent, Aldrich, Germany) for 30 min to remove the remaining metal impurities and then extensively washed with deionized water to achieve a neutral pH. The resulting material was dried in a drying oven at 90 °C for 24 h. The yield of porous carbon synthesized from pre-carbonized MW pappus was 50%. The porous carbon prepared in this manner is denoted as milkweed pappus carbon (MPC).

### 2.2. Synthesis of Milkweed Pappus Carbon–Sulfur (MPC–S) Composites

MPC–sulfur composites with different sulfur contents were prepared using a wet-impregnation method [34]. Wet impregnation was carried out between MPC and sulfur as follows: 0.3 g MPC was added to an optimum amount of a S/CS_2_ solution (0.3 g S/5 mL CS_2_) at different carbon/sulfur weight ratios (3:5, 1:4, and 1:15) with magnetic stirring for 1 h. A black colored solution was obtained, which was impregnated into MPC followed by air drying in a fume hood. The subsequent sample was transferred into a Teflon-lined autoclave vessel in an air atmosphere. The vessel was heated at 155 °C for 12 h in the atmosphere. The obtained products were denoted as milkweed pappus carbon-sulfur (MPC-S) composites.

### 2.3. Characterizations

The Brunauer–Emmett–Teller (BET) surface area and Barret–Joyner–Halenda pore-size distribution of the samples were measured at 77 K (ASAP 2020 Analysis, Micromeritics Instruments, Norcross, GA, USA). The powder X-ray diffraction (XRD, PANalytical Malvern, Malven, UK) patterns of all samples were recorded on Philips X’Pert (X’Pert^3^, PANalytical Malvern, Malven, UK) Pro equipped with Cu-Kα radiation (λ = 1.5418 Å) for 2θ = 10–70°. Raman spectroscopy was performed using an XperRam 200 (Nano Base Inc., Korea) system with 532 nm diode laser excitation on a 300 lines mm^−1^ grating. The sulfur content of the samples was determined via thermogravimetric analysis (TGA, TA Q600–0825, TA Instruments, DE, USA). The temperature was increased to 800 °C at a rate of 10 °C min^−1^ under Ar flow. In addition, the sulfur content of the samples was determined using an elemental analyzer (EA, Vario Micro Cube, Elementar, Germany). The surface and cross-sectional morphologies of the samples were obtained using high-resolution scanning electron microscopy (SEM, SU70, Hitachi, Japan). The interior structures of the MPC–S composites were observed using transmission electron microscopy (JEM 2100F, Jeol, Japan). The surface chemical compositions of the samples were analyzed by performing X-ray photoelectron spectroscopy (XPS, Thermo Corporation K-Alpha, USA) using a Thermo Electron Corporation spectrometer with Al Kα (1486.6 eV) radiation at a spot diameter of 400 mm with charge compensation. The emitted photoelectrons are detected using a multichannel detector at a take-off angle of 90 with respect to the sample surface. During the measurement, the base pressure in the turbo-pumped analysis chamber is maintained at 1.2 × 10–9 mbar. Survey spectra are acquired at a pass energy of 200 eV and resolution of 1 eV, while high-resolution spectra are acquired at a pass energy of 50 eV and resolution of 0.1 eV.

### 2.4. Electrochemical Measurements

The electrochemical measurements of the MPC–S composites were conducted using CR2032 coin-type cells assembled in an Ar-filled glove box with Li foil as the counter electrode. A working electrode was prepared by mixing the as-synthesized active material (MPC–S composite, 70 wt.%), conductive material (Denka Black, Li-250, Singapore), and polymeric binder (polyvinylidene fluoride 2.5 wt.% dissolved in *N*-methyl-2-pyrrolidone, 10 wt.%; Solef^®^ 5130, Solvay, Belgium) in *N*-methyl-2-pyrrolidone. The resultant slurry was spread on the surface of an aluminum foil current collector and then dried at 80 °C for 2 h and then roll-pressed.

The other MPC-S composite cathodes were prepared in the same manner, where the active material, conductive material, and PVdF binder were mixed in a weight ratio of 70:20:10.

The resulting electrode thickness was approximately ~20 μm, and mass loading of an active material was approximately ~1.0 mg cm^−2^. The separator was a Celgard 2400 polypropylene (PP) membrane, and the electrolyte was 1.0 M lithium bis(trifluoromethanesulfonyl)imide and 0.4 M LiNO_3_ in a solvent mixture of dioxolane and dimethoxyethane (1:1 vol.%). Electrochemical cycling and rate tests were performed in a cut-off voltage range of 1.5–2.8 V versus Li/Li^+^. CV was performed at a scan rate of 0.05 mV s^−1^ using a galvanostatic/potentiostatic system (WonATech Co., Ltd., Seoul, Korea). Electrochemical impedance spectroscopy (EIS) measurements were conducted using a ZIVE SP2 analyzer (WonATech Co., Ltd., Korea) in a frequency range of 1–10 mHz with an AC amplitude of 10 mV.

## 3. Results

**Figure 1:**
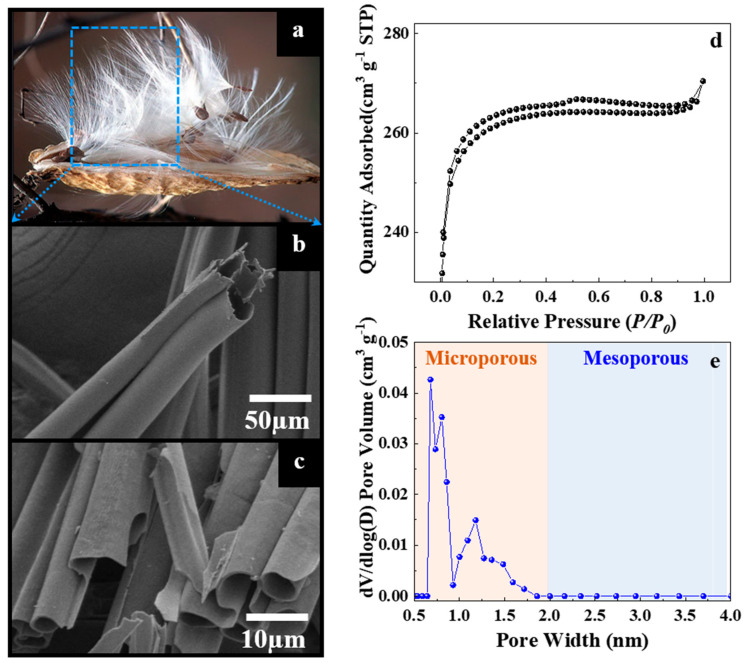
(**a**) Photograph and (**b**) SEM image of the actual MW pappus. (**c**) SEM image after carbonization. (**d**) Nitrogen adsorption-desorption isotherms. (**e**) Pore size distribution of the MPC sample.

Figure 1a shows white fibers of the actual MW pappus. The pappus contains a regular circular hole at the center with a diameter of 17–20 μm, as shown in the SEM image (Figure 1b). After carbonization, the average diameter of the tubes is reduced to 3.3 μm. but MPC maintains the structure of a straight tube with smooth walls (Figure 1c). The specific surface area and pore structure of the MPC sample were determined from nitrogen adsorption–desorption isotherms. As shown in Figure 1d, the isotherms are of type I based on the IUPAC classification. The knee of the isotherms occurs at an extremely low relative pressure (P/P_0_ < 0.05). Furthermore, the plateau is flat, indicating the presence of highly microporous carbon [37]. The pore size distribution curve proves that the MPC sample mainly has a microporous size (<2 nm), which is beneficial for restricting the dissolution of polysulfides and facilitating the fast migration of Li ions (Figure 1e). In view of the micropore size only small sulfur molecules can be accommodated in the micropores of MPC, while the large sulfur molecules cannot be stored. By using smaller sulfur molecules (S_2–4_), the aim is to confine them in the confined space of a conductive microporous carbon matrix as the starting active material [38,39]. The BET surface area, total pore volume, and pore width of the MPC sample are 1056 m^2^ g^−1^, 0.48 cm^3^ g^−1^, and 0.75–1.25 nm, respectively.

**Figure 2:**
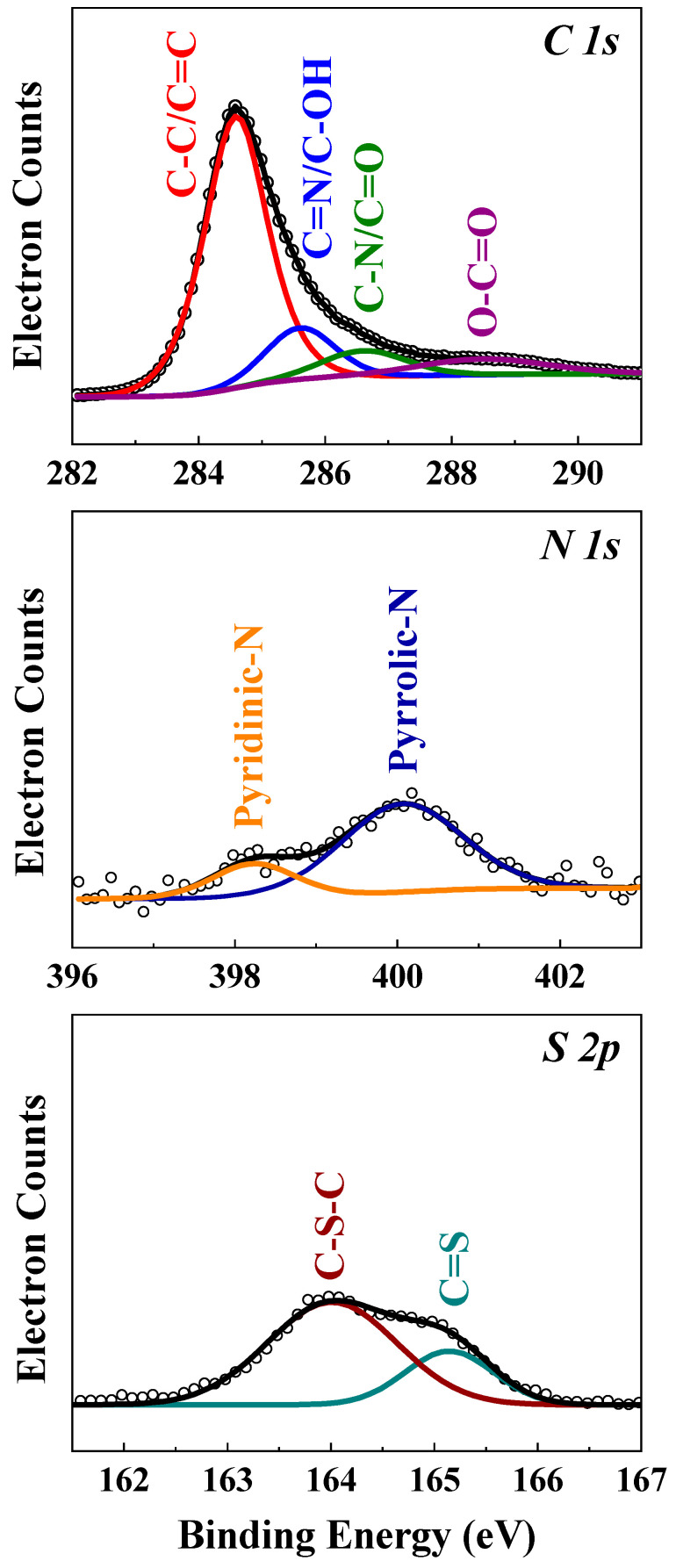
XPS spectra of the MPC sample for C 1s, N 1s, and S 2p.

The surface chemical components of MPC were evaluated using XPS. The high-resolution C 1s, N 1s, and S 2p XPS spectra of MPC are presented in Figure 2. The deconvolution of the C 1s carbon and oxygen atoms is observed. These four peaks are related to C=C-C bonds (284.7 eV), C-O bonds (286.4 eV), C=O bonds (287.7 eV), and O-C=O bonds (289.7 eV) [40]. The fractions of C=C, C-O, C=O, and O-C=O in the in MPC sample are as high as 73.9 at.%, 7.8 at.%, 9.5 at.%, and 8.8 at.%, respectively, indication a significant amount of oxygen. The oxygen content was examined using an elemental analyzer (Table S1). The N 1s spectrum shows two peaks at 398.5 eV and 400.6 eV. This is due to the pyridinic-N, pyrrolic-N, and N oxides present in the carbon structure, as discussed in the literature [27,41].

A weak broad peak centered at 169.4 eV is observed for MPC, which is likely due to the surface oxidation of sulfur or the strong interaction between sulfur and MPC. The peaks at 164.1 eV and 165.3 eV can be assigned to the remaining sulfur. The peak at 164.5 eV is attributed to S-O. This interaction may have an adsorbing ability to anchor S atoms and prevent the subsequently formed polysulfides from dissolving in the electrolyte during cycling [42].

**Figure 3:**
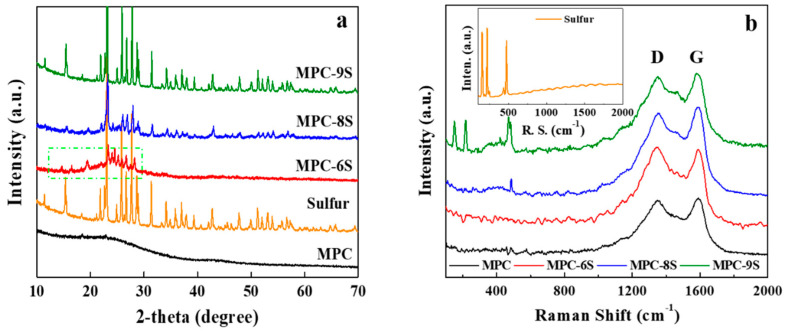
(**a**) XRD patterns and (**b**) Raman spectra of MPC, elemental sulfur, and MPC-sulfur composites with different sulfur contents (inset: Raman spectrum of elemental sulfur).

The XRD patterns of sulfur, as prepared MPC, MPC-6S, MPC-8S, and MPC-9S are shown in Figure 3a. There is a broad peak at a 2θ = ~24°, which corresponds to the 002-plane reflection of graphite. In addition, there is a small peak at 2θ = ~44°, which corresponds to the 100-plane reflection of graphite. These two broadening peaks reveal the possible presence of an amorphous phase and pseudo–graphite within carbonaceous MPC-6S, MPC-8S, and MPC-9S [43]. All XRD patterns of sulfur, MPC-6S, MPC-8S, and MPC-9S exhibit orthorhombic structures for elemental sulfur. Compared with sulfur, MPC-6S, MPC-8S, and MPC-9S show sharp peaks of sulfur with increased peak intensity. This is because sulfur is well dispersed within the carbon nanopores. In addition, this indicates that the amount sulfur that is attached to the surface of MPC increases with the sulfur content.

The Raman spectra of MPC, MPC-6S, MPC-8S, and MPC-9S are shown Figure 3b. There are two peaks at 1360 cm^−1^ and 1585 cm^−1^, which represent the D and G bands, respectively. The G-band peak at approximately 1580 cm^−1^ arises from a bond stretching vibration corresponding to graphitic carbon, whereas the D-band peak at 1350 cm^−1^ is ascribed to disorders and defects [44]. The typical peaks located at about 1590 and 1355 cm^−1^ clearly correspond to the G band from the ordered structure of graphitic crystallites and the D band resulting from the properties of lattice defects and disorder, respectively [45]. The peak intensity ratio (I_G_/I_D_) of the MPC is calculated to be 1.072, which is higher than those of the reported S/porous carbon composites as shown in Figure 6 and Table 1 [46]. The I_G_/I_D_ indicates the degree of graphitization of carbon, which is an essential condition for improving the conductivity of carbon materials, and it helps facilitate the electron transport of sulfur in the electrochemical process. The decremental I_G_/I_D_ values of the MPC-S composites indicates that more defects emerge after sulfur infusion [47].

The D and G bands appear in the spectra of the MPC and MPC-S composites, and sulfur is well dispersed inside MPC compared to the surface. The nanosulfur powders show strong peaks at 163 cm^−1^, 229 cm^−1^, and 483 cm^−1^. In addition, MPC-9S exhibits three peaks at less than 500 cm^−1^, which are due to sulfur particles. However, the peaks are weak in the case of MPC-8S and MPC-6S. The energy dispersive spectroscopy (EDS) mapping shown in Figure S2 is almost consistent with the fact that elemental sulfur is less distributed on the MPC surface and abundant in the tubular structure [48].

Elemental analysis and TGA were performed to determine the contents of elemental carbon, oxygen, and sulfur in the sulfur-loaded composites. (Table S1, Figure S1) The sulfur contents of MPC-6S, MPC-8S, and MPC-9S are 58.47 wt.%, 77.79 wt.%, and 91.33 wt.%, respectively. These are similar to the actual sulfur contents of the MPC-S composites (62.5 wt.%, 80.0 wt.%, and 93.8 wt.%) that are input during synthesis. The carbon contents of MPC, MPC-6S, MPC-8S and MPC-9S are 79.40 wt.%, 34.51 wt.%, 17.13 wt.%, and 7.33 wt.%, respectively. This is because a large amount of sulfur fills the spaces in the pores of carbon, and the oxygen content decreases. The sulfur content obtained using TGA is almost consistent with that obtained using elemental analysis. The morphologies of MPC-6S, MPC-8S, and MPC-9S were characterized using SEM, as shown in Figure S2a. The composites are encapsulated with sulfur in the tubular structure with micrometer-sized diameters. In particular, MPC-9S is filled with embedded sulfur in the tubular structure. This is because high amounts of sulfur are present in the MPC-S composites, which can fill the pores or become attached to the outer surface. Figure S2b shows the high-resolution transmission electron microscopy images of MPC-6S, MPC-8S, and MPC-9S. The EDS mapping for sulfur and carbon is shown in Figure S2b. The EDS mapping shows that the distributions of carbon (green) and sulfur (yellow) are slightly different. The distribution of sulfur in the synthesized MPC-S composites is concentrated on the tubular surface and in the tubular structure. This can improve cyclability by suppressing the dissolution of Li_x_S during the charging and discharging processes.

**Figure 4:**
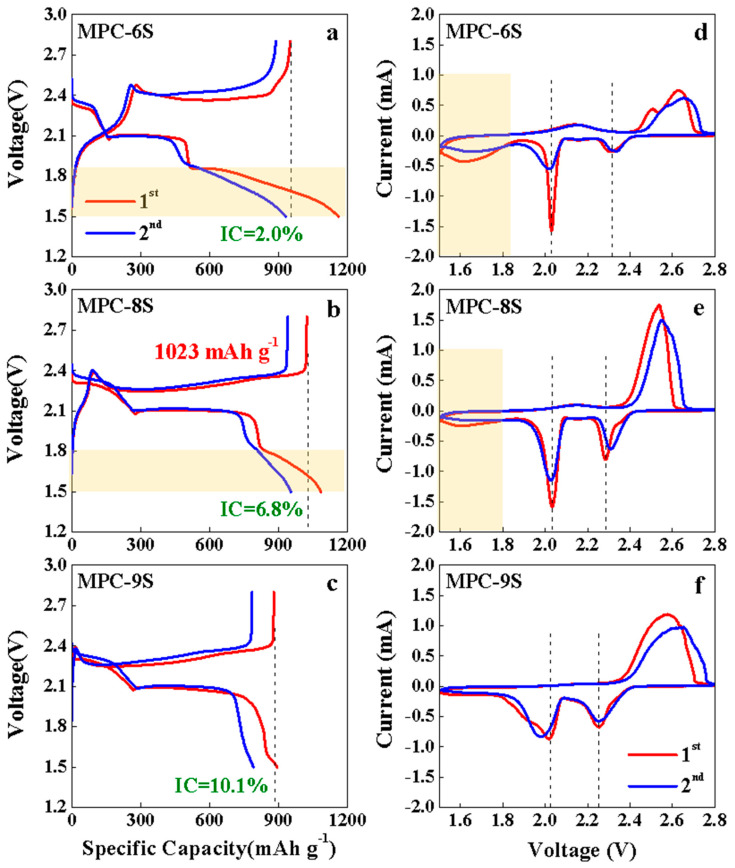
(**a**–**c**) Charge-discharge voltage profiles (1st and 2nd) and (**d**–**f**) cyclic voltammograms of the MPC-6S, MPC-8S, and MPC-9S composite cathodes at 0.1 C.

Figure 4a–c shows the 1st and 2nd charge–discharge voltage profiles of the MPC-6S, MPC-8S and MPC-9S cathodes. The initial charge/discharge capacities of the MPC-6S, MPC-8S and MPC-9S cathodes are 950 mAh g^−1^/931 mAh g^−1^, 1023 mAh g^−1^/953 mAh g^−1^, and 879 mAh g^−1^/790 mAh g^−1^, respectively. The irreversible capacity increases with the sulfur content, and the MPC-6S cathode shows the lowest at 2.0%. The MPC-9S cathode exhibits two normal plateaus, while the MPC-6S and MPC-8S cathodes exhibit three distinct potential regions. These signatures correspond to the formation of long-chain soluble polysulfides in the first region at 2.3 V and short-chain solid sulfides in the second region at 2.07 V and loss of redox ability in the third region at 1.80 V. The potential hysteresis phenomenon is observed in the third region, which could be attributed to the extra electrode polarization required to overcome the confinement barrier of the high adsorption energy [49,50,51]. This is also confirmed by the peak observed at 1.6–1.8 V in the CV results. In addition, the peak observed at 2.4 V during discharging moves to the left as the sulfur content increases. Thus, this peak is also due to carbon. CV measurements were conducted to investigate the electrochemical mechanisms of the as-prepared Li-S batteries.

Figure 4d,f shows the CV curves of the MPC-6S, MPC-8S, and MPC-9S electrodes at a scan rate of 0.1 mV s^−1^ during the first two cycles. The CV curves of the Li/S cell with the MPC-6S, MPC-8S, and MPC-9S cathodes are shown in Figure 4d,e. The CV data provide evidence for two redox processes for sulfur reduction and oxidation in the system, which agrees well with literature [52]. There are three peaks in the first cathodic reduction process. The peak at 2.28 V (vs. Li^+^/Li^0^) corresponds to the reduction in elemental sulfur (S_8_) to polysulfide anions (S_x_^2−^; 2 < x < 8). A strong cathodic peak at 2.03 V (vs. Li^+^/Li^0^) suggest a strong reduction in soluble polysulfide anions to an insoluble low-order Li_2_S_2_/Li_2_S deposit. The peaks at 2.28 V for the MPC-6S and MPC-8S in second cathodic reduction process shift to higher reduction potential of 2.31 V. The improved kinetics is caused by the conductive microporous structure that minimizes the barrier of electron transfer and lithium ion migration. The MPC-8S and MPC-9S cathodes show two overlapping oxidation peaks at 2.53–2.57 V, while the MPC-6S cathode exhibits distinct peaks. During the anodic oxidation of lithium sulfides to polysulfides, partial unconstrained dissolution of polysulfide ions causes a reduction in the anodic current, which is stabilized at second cycle.

In the 1st CV profile of the MPC-8S cathode, the oxidation/reduction peak is considerably narrower than those of the MPC-6S and MPC-9S cathodes. Furthermore, the MPC-8S cathode shows a more uniform sulfur distribution. The voltage at which the oxidation/reduction peak appears in the CV results is consistent with the voltage plateau observed during charging and discharging in Figure 4a–c.

**Figure 5:**
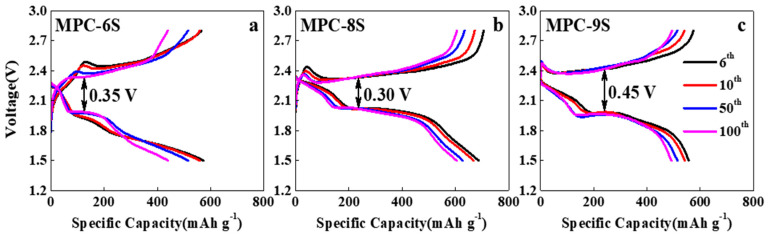
(**a**–**c**) Charge-discharge voltage profiles of the MPC-6S, MPC-8S and MPC-9S cells after cycling.

Figure 5a–c shows the voltage profiles up to 100 cycles. There is a clear difference in the voltage hysteresis between charging and discharging at the average capacity of the different samples. The voltage hysteresis of MPC-8S is lower (0.30 V) than those of the other two composites. The charge/discharge profiles of MPC-6S and MPC-9S change as the charge/discharge cycle continues. The low polarization of MPC-8S may be the result of a more uniform sulfur dispersion in MPC. This results in more intimate contact between carbon and sulfur, thereby reducing the charge resistance and delaying the dissolution of lithium polysulfide into the electrolyte. This is consistent with the result that the discharge capacity of MPC-8S is higher than those of MPC-6S and MPC-9S.

**Figure 6:**
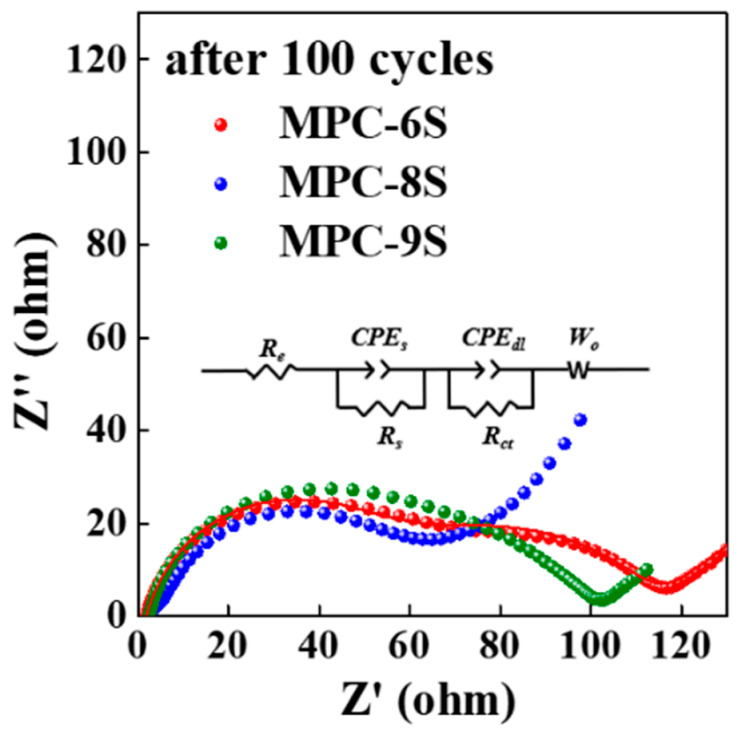
EIS results and equivalent circuit model of the MPC-6S, MPC-8S, and MPC-9S cells after 100 cycles.

The electrochemical characteristics of the MPC-S composites were examined by performing EIS after 100 cycles, and the results are shown in Figure 6 and Table 2. Before the electrochemical reaction, the EIS spectra of the composite cathodes are composed of one depressed semicircle in the high-frequency region (R_ct_) and a short inclined line (Warburg impedance) in the low-frequency region [11]. After 100 cycles, the EIS spectra of the three samples in the fully charged state exhibit two depressed semicircles followed by a long sloping line. The semicircle in the high-frequency region represents the interfacial charge transfer process (R_ct_), and the semicircle in the medium-frequency region can be attributed to the passivation film formed by the irreversible redeposition and aggregation of lithium polysulfide on the electrode surface (R_s_) [53]. The value of R_ct_ for the MPC-8S cathode is smaller than those for the other cathodes. This implies that sulfur is well dispersed inside MPC, and the internal resistance is the lowest. After 100 cycles, R_ct_ is approximately 60.1 Ω for MPC-8S, and it is similar for MPC-6S (81.2 Ω) and MPC-9S (83.5 Ω). In the case of MPC-8S, one semicircle with almost no R_s_ is observed. This implies that the electrode processes do not increase the resistance of the interfacial passivation films by irreversible redeposition, and the aggregation of lithium polysulfide on the electrode surface is less than that for the other composite cathodes. This supports the result that sulfur dispersion and encapsulation are maintained better in MPC-8S compared to the other composites.

**Figure 7:**
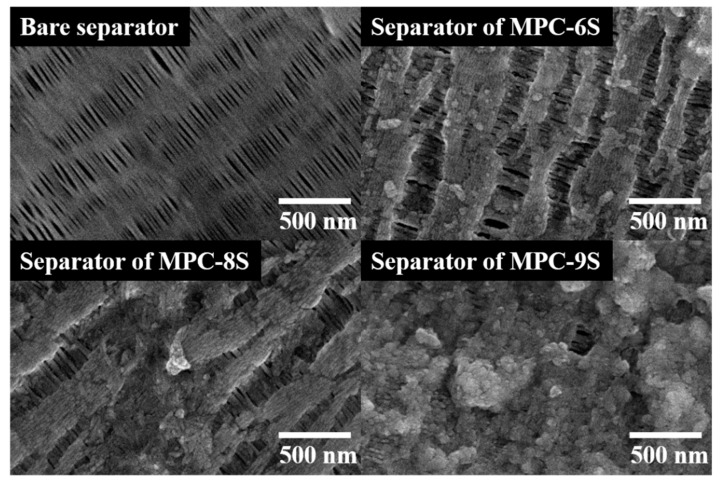
Surface images of the bare separator and separators of MPC-6S, MPC-8S, and MPC-9S after 100 cycles.

Figure 7 shows the SEM images of the separators after dismantling the MPC-6S, MPC-8S, and MPC-9S composite cathodes after the 100th cycle. During the charge and discharge processes, lithium polysulfide dissolved in an organic solvent can freely move through the pores of the separator and reach the lithium anode. Li_x_S is deposited on the surface of the separator owing to continued charging and discharging. In the case of MPC-9S, the largest amount of lithium polysulfide is dissolved, and it blocks the separator, thereby reducing the capacity.

**Figure 8:**
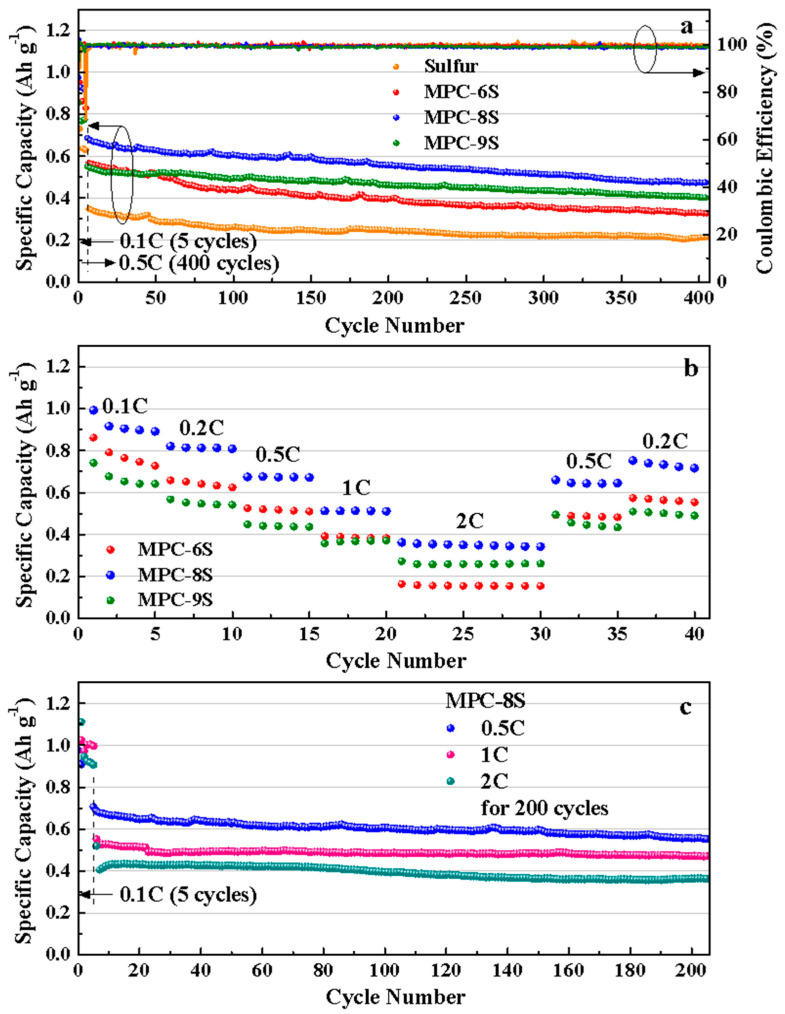
(**a**) Cycling performance and (**b**) Rate capability of the MPC-6S, MPC-8S, and MPC-9S cells. (**c**) Cycling performance of the MPC-8S at 0.5C, 1C, 2C for 200 cycles.

Figure 8a shows the comparison of the charging and discharging characteristics of the MPC-6S, MPC-8S, and MPC-9S cathodes up to 400 cycles. To prevent the capacity degradation due to a large increase in current, the initial 5 cycles were performed at 0.1 C and the subsequent cycles at 0.5 C. The capacity decreases as the current density increases. This due to the structural and interfacial stabilization of Li-S batteries or a small amount of residual sulfur on the carbon surface; however, further investigation is required [54,55]. The capacity retention rate of MPC-8S is 81% at 556 mAh g^−1^ even after 200 cycles, and it has the highest discharge capacity after 400 cycles. This shows that sulfur is best dispersed in MPC with a sulfur content of 80%, and it maintains a structure that prevents dissolution of polysulfides. MPC-9S shows rapid capacity reduction, and it has the lowest discharge capacity at 401 mAh g^−1^ after 400 cycles. The capacity decreases because the dissolution rate of polysulfides increases at a high sulfur content, which is abundant inside and on the surface of MPC. Figure 8b shows the C rate characteristics. Capacity retention occurs at high rates of 0.1 C–2 C and capacity recovery at 0.5 C. MPC-8S shows the best capacity from 0.1 C to 2 C and the best recovery ability at 0.2 C. MPC-9S shows the smallest decrease in capacity at a high C rate. However, the capacity decreases owing to the dissolution of polysulfides. This can be prevented by coating the separator with carbon flakes; this is described later. Figure 8c shows the cycle characteristics of the MPC-8S composite cathodes up to 200 cycles at 0.5 C, 1 C, and 2 C. The discharge capacities of the cathodes are 590 mAh g^−1^, 500 mAh g^−1^, 400 mAh g^−1^ at 0.5 C, 1 C, and 2 C up to 200 cycles, respectively.

We compared the data reported in several previous papers (Table 3) and found that the data for milkweed pappus is either slightly better than or similar to those for reported materials. This excellent performance of MPC material may be due to its micropores/mesopores porous structure, which supports the dissolution of polysulfides, helps the electrolyte infiltration and increases the number of active sites.

**Figure 9:**
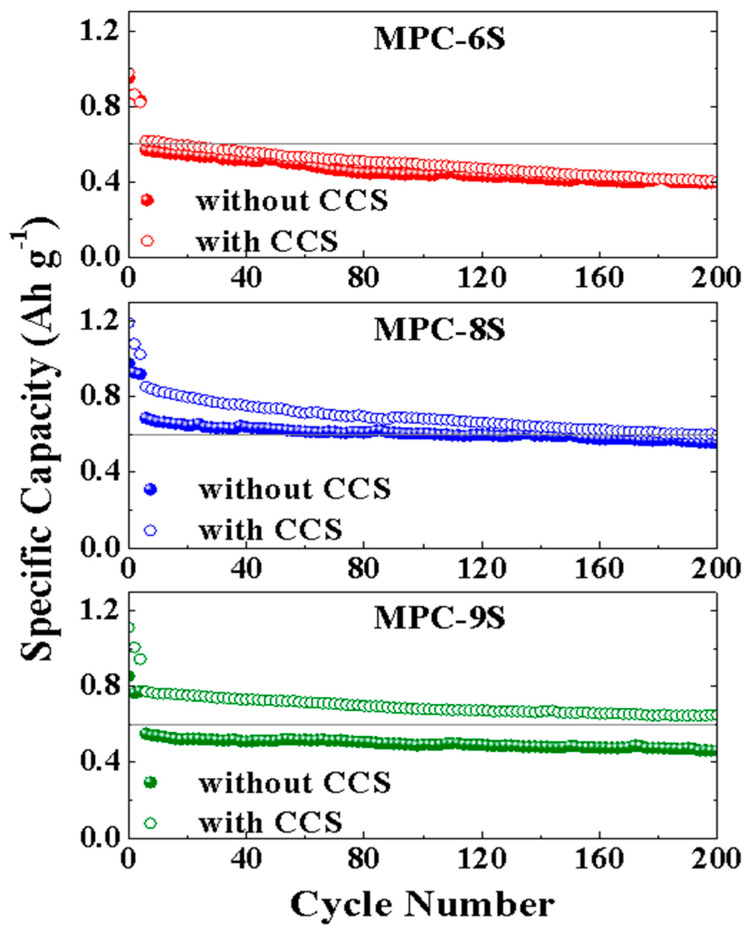
Cycling performance of the MPC-6S, MPC-8S, and MPC-9S cells coupled with PP/CCS separator.

Figure 9 shows the results of 200 charging and discharging cycles of MPC-6S, MPC-8S, and MPC-9S cells with and without the PP/CCS(carbon coated separator). The capacity of MPC-9S with the CCS improves from 463 mAh g^−1^ to 743 mAh g^−1^ after 200 cycles, and it shows the best capacity retention rate. There is no significant change in the capacity of MPC-6S even with the CCS. This implies that the contact with conductive carbon is not smooth and not that the capacity decreases because of the dissolution of lithium polysulfide. The CCS was fabricated by coating the flat surface of the separator with thin flake graphite particles. The CCS surface consists of closely packed thin flake-type graphite particles. The thin multilayer graphite film prevents lithium polysulfide from moving through the PP separator. Moreover, the CCS acts as a mechanical barrier to the diffusion of lithium polysulfide from the anode into the electrolyte, which improves the sulfur utilization and capacity [56].

To design a Li-S battery with a higher energy density compared to current lithium-ion batteries, the loading mass of sulfur at the positive electrode and thus the specific area capacity should be more than 2 mg cm^−2^ and 2 mAh cm^−2^, respectively. A recent study reported an increase in the loading mass of a pure sulfur anode combined with a carbon layer acting as the top current collector [57].

**Figure 10:**
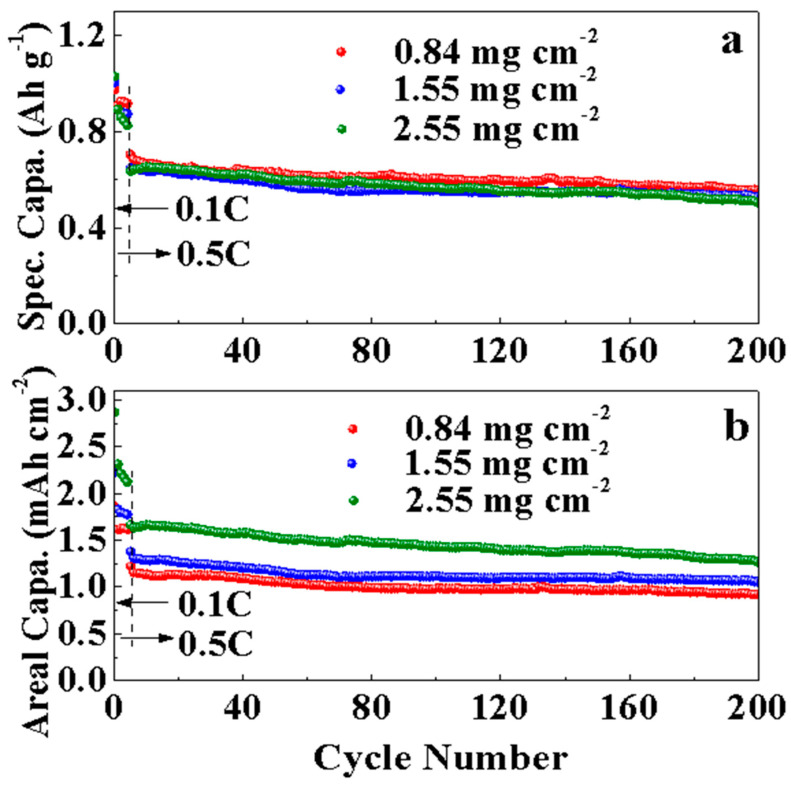
(**a**) Specific capacities and (**b**) areal capacities of the MPC-8S cell with different mass loading.

Figure 10 shows a loading mass of 2.55 mg cm^−2^, which is approximately three times higher than the loading mass of MPC-8S obtained earlier (0.84 mg cm^−2^). As the thickness of the electrode with a high loading mass increases, its initial capacity becomes more similar to that of the thin electrode. However, the fading speed slightly increases, and the cycle characteristics are not good. This phenomenon does not occur smoothly in the insertion/desorption of lithium because of poor contact between sulfur and conductive carbon and thickness. This problem will be addressed in future work.

## 4. Conclusions

Porous carbon was prepared from MW pappus to improve the performance of Li-S batteries. Carbon–sulfur composites were prepared using the synthesized porous carbon to increase the electrical conductivity of sulfur and to prevent dissolution of lithium polysulfide while encapsulating sulfur in the porous carbon. The MW pappus was carbonized and activated to obtain space for sulfur impregnation into MPC. MPC was loaded with sulfur at contents of 62.5%, 80.0%, and 93.8%, and its physical and electrochemical properties were evaluated. MPC-8S (80% sulfur) exhibited the best capacity and charge/discharge cycling characteristics. It showed the highest discharge capacity and excellent cycle stability even after 400 cycles. The loading mass is an important parameter in the design of Li-S batteries with a higher energy density compared to state-of-the-art lithium-ion batteries. Life characteristics were observed by increasing the loading mass to 2.5 mg cm^−2^, which was approximately three times higher than that of MPC-8S. However, the fading speed slightly increased and the cycle characteristics were poor. The capacity of MPC-9S was significantly reduced because of the high sulfur content. The FCS was used to control the free movement of lithium polysulfide through the pores of the separator during the charge and discharge processes. It acted as a barrier between the positive electrode and separator to prevent polysulfide diffusion and increase the discharge capacity. The capacity of MPC-9S with the FCS improved from 463 mAh g^−1^ to 743 mAh g^−1^ after 200 cycles, confirming its excellent capacity retention.

## Figures and Tables

**Table 1:** Sulfur content and I_G_/I_D_ ratio of the MPC, MPC-S composites.

	Sulfur Content [wt%]	I_G_/I_D_
MPC	-	1.072
MPC-6S	54.47	1.036
MPC-8S	77.79	1.029
MPC-9S	91.33	0.971

**Table 2:** Values of Rs and Rct for the MPC-6S, MPC-8S, and MPC-9S cells after 100 cycles.

	Rs (Ω)	Rct (Ω)
MPC-6S	1.74	81.2
MPC-8S	1.37	60.1
MPC-9S	2.15	83.5

**Table 3:** Cathode materials and their properties for Li-S battery.

Material for Carbonization	Activation Temperature (°C)	Surface Area (m^2^ g^−1^)	Pore Volume (cm^3^ g^−1^)	Capacity (mAh g^−1^)	Ref.
Banana peels	900	194	2.40	C dis = 832.4 mAh g^−1^ Cycle number = 200 Rate = 0.2 C	[24]
Bamboo leaves	800	329	0.5	C dis = 707 mAh g^−1^ Cycle number = 200 Rate = 1 C	[25]
Soybeans	800	1500	0.7	C dis = 460 mAh g^−1^ Cycle number = 800 Rate = 0.5 C	[26]
Corn cobs	800	2724	1.46	C dis = 720 mAh g^−1^ Cycle number = 150 Rate = 0.3 C	[27]
Pomelo peels	600	1533	0.837	C dis = 750 mAh g^−1^ Cycle number = 100 Rate = 0.2 C	[28]
Shaddock peels	900	937.1	0.82	C dis = 619.8 mAh g^−1^ Cycle number = 100 Rate = 0.5 C	[29]
Poplar catkins	800	186	0.287	C dis = 810 mAh g^−1^ Cycle number = 100 Rate = 0.1 C	[30]
Kapok fibers	700	282.38	0.1574	C dis = 524 mAh g^−1^ Cycle number = 90 Rate = 0.4 A g^−1^	[31]
Milkweed pappuss	800	1056	0.48	C dis = 743 mAh g^−1^ Cycle number = 200 Rate = 0.5 C	This work

## Data Availability

The data is available on reasonable request from the corresponding authors.

## Supplementary Materials

The following supporting information can be downloaded at: https://www.mdpi.com/article/10.3390/nano12203605/s1, Figure S1: The TGA profiles used to determine the sulfur contents in the sulfur-loaded samples.; Figure S2: SEM images (a) and HR TEM images and EDS mapping Showing Distribution of C, S and N (b) of MPC-6S, MPC-8S and MPC-9S powders; Table S1: Elemental content of the MPC and MPC-sulfur composites in EA.
