# Supplementary material for: Synthesis and Electrochemical Performance of Microporous Hollow Carbon from Milkweed Pappus as Cathode Material of Lithium–Sulfur Batteries
Source: Nanomaterials (Basel). 2022 Oct 14;12(20):3605. doi: 10.3390/nano12203605 (PMC9606866; doi:10.3390/nano12203605)
Supplement: Supplementary file 1 [file nanomaterials-12-03605-s001.zip › nanomaterials-1954111-supplementary.pdf]

Supporting Information

Synthesis and Electrochemical Performance of Microporous Hollow Carbon from Milkweed Pappus as Cathode Material of Lithium–Sulfur Batteries

Jun-Ki Kim [1], Yunju Choi [1], Euh Duck Jeong [1], Sei-Jin Lee [2], Hyun Gyu Kim [1], Jae Min Chung [3], Jeom-Soo Kim [4], Sun-Young Lee [5,*,†] and Jong-Seong Bae [1,*,†]

1 Busan Center, Korea Basic Science Institute (KBSI), Busan 46742, Korea
2 Jeonju Center, Korea Basic Science Institute (KBSI), Jeonju 54907, Korea
3 Division of Plant Resources, Korea National Arboretum, Seoul 02455, Korea
4 Department of Chemical Engineering, Dong-A University, Busan 49315, Korea
5 Secondary Batteries Technology Center, Chungnam Techno Park, Cheonan 31035, Korea
* Correspondence: sylee22@ctp.or.kr (S.-Y.L.); jsbae@kbsi.re.kr (J.-S.B.)
† These authors contributed equally to this study.

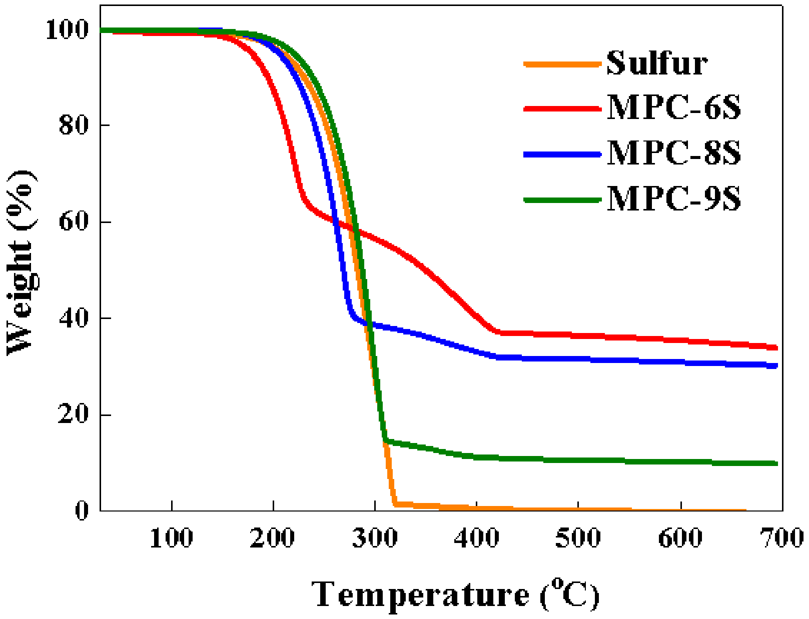

Figure. S1. The TGA profiles used to determine the sulfur contents in the sulfur-loaded samples.

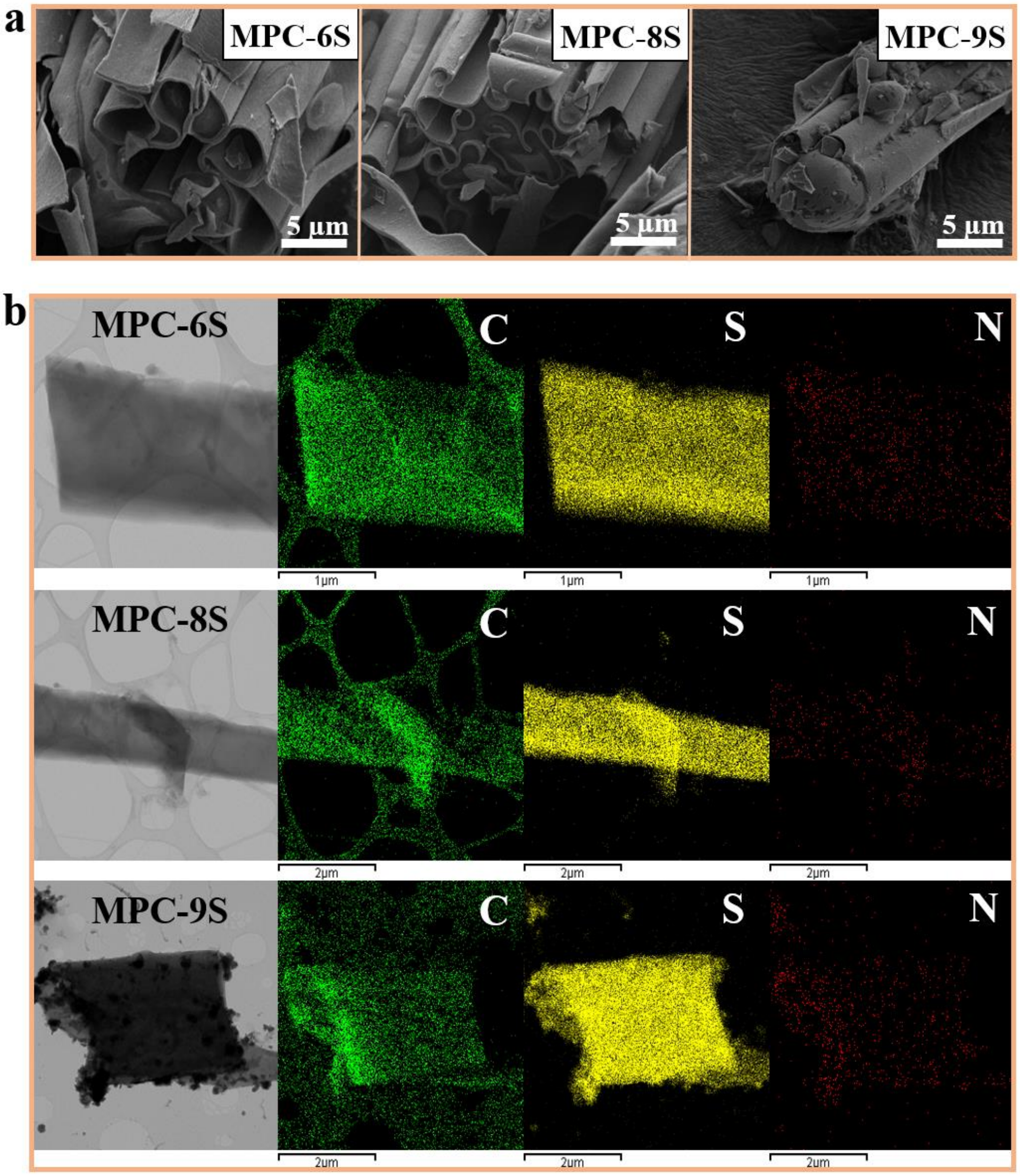

Figure S2. SEM images (**a**) and HR TEM images and EDS mapping Showing Distribution of C, S and N (**b**) of MPC-6S, MPC-8S and MPC-9S powders.

Table S1. Elemental content of the MPC and MPC-sulfur composites in EA.

	**C (wt.%)**	**H (wt.%)**	**N (wt.%)**	**S (wt.%)**	**O (wt.%)**
MPC	79.40	2.22	0.01	0.30	18.07
MPC-6S	34.51	0.29	0.30	58.47	6.43
MPC-8S	17.13	0.30	0.36	77.79	4.42
MPC-9S	7.33	0.37	0.29	91.33	0.68
